# HPV vaccination leads to decrease of anogenital warts and precancerous lesions of the cervix uteri in young women with low vaccination rates: a retrospective cohort analysis

**DOI:** 10.1186/s12885-022-10214-1

**Published:** 2022-12-09

**Authors:** Vanesa Osmani, Sandra Fett, Martin Tauscher, Ewan Donnachie, Antonius Schneider, Stefanie J. Klug

**Affiliations:** 1 Chair of Epidemiology, Department of Sport and Health Sciences, Technical University of Munich, Georg-Brauchle-Ring 56, 80992 Munich, Germany; 2 The Bavarian Association of Statutory Health Insurance Physicians (KVB), Elsenheimerstraße 39, 80687 Munich, Germany; 3 Institute of General Practice and Health Services Research, TUM School of Medicine, Technical University of Munich, Orleansstrasse 47, 81667 Munich, Germany

**Keywords:** HPV, Vaccination, Anogenital warts, Precancerous lesions, Cervix uteri, Prevention

## Abstract

**Background:**

Although the human papillomavirus (HPV) vaccine has been recommended in Germany for girls since 2007, no organised vaccination programme was introduced and HPV vaccine coverage remains low. We investigated the HPV vaccination rates from 2008 to 2018 and the effects of HPV vaccination on anogenital warts and precancerous lesions in young women in Bavaria, Germany, a state with low vaccination rates.

**Methods:**

Retrospective analyses of claims data from the Bavarian Association of Statutory Health Insurance Physicians (KVB) on females born between 1990 and 2009 (9 to 28 years old in 2018) were conducted to calculate vaccination rates by birth cohort, proportion of vaccine types administered and incidence of anogenital warts and precancerous lesions of the cervix uteri. 942 841 Bavarian females 9 to 28 years old with available information on HPV vaccination were included to calculate vaccination rates. For the outcome analyses, data from 433 346 females 19 to 28 years old were analysed. Hazard ratios (HR) were computed from univariable and multivariable Cox regression models comparing vaccinated and unvaccinated women, considering type of vaccine used and contraceptive prescription.

**Results:**

40·9% of 18-year-olds and only 13·3% of 12-year-olds were fully vaccinated in 2018 in Bavaria. Gardasil® and Gardasil9® were most commonly administered. Vaccinated compared to unvaccinated women had a lower incidence of anogenital warts and cervical lesions, however only small differences were detected between fully and partially vaccinated women. Fully vaccinated women had a 63% (HR 0·37 (95% confidence interval (CI) 0·34 to 0·40) and 23% (HR 0·77, 95%CI 0·71 to 0·84) lower risk of anogenital warts and cervical lesions, respectively. Women who were prescribed contraceptives prior to vaccination had a 49% higher risk of developing anogenital warts (HR 1·49, 95%CI 1·25 to 1·79) or cervical lesions (HR 1·49, 95%CI 1·27 to 1·75) compared to vaccinated women without contraceptive prescription.

**Conclusions:**

The evaluation of the effects of HPV vaccination in Bavaria showed a promising decline of anogenital warts and precancerous lesions in vaccinated young women. However, an increase in vaccination rates is necessary to achieve a greater population impact in preventing HPV-related diseases.

**Supplementary Information:**

The online version contains supplementary material available at 10.1186/s12885-022-10214-1.

## Introduction

Cervical cancer is one of the leading causes of morbidity and death in women worldwide [1, 2]. High-risk (hr) human papillomaviruses (HPV) are sexually transmitted viruses, which are causally associated with the development of cervical cancer [3]. Persistent infections with 12 hrHPV types (16, 18, 31, 33, 35, 39, 45, 51, 52, 56, 58, and 59) are responsible for almost all cervical cancers [1, 4]. In infected individuals, most HPV infections clear within two years, with less than 10% developing into persistent infection, and potentially precancerous lesions [5]. Low-risk (lr) HPV types 6 and 11 are associated with approximately 90% of anogenital warts [6]. The yearly incidence of anogenital warts in both males and females ranges from 160 to 289 per 100,000 worldwide [7].

Since 2006, HPV vaccination has been introduced in many countries globally to prevent HPV infection. The HPV vaccines are considered highly effective and safe, and should be preferably administered before the first sexual contact [8, 9]. Three different vaccines were approved by the FDA (Food and Drug Administration, USA) and EMA (European Medicines Agency, Europe): Gardasil® (Merck, Sharp & Dohme Corp.), Gardasil9® (Merck, Sharp & Dohme Corp.), and Cervarix® (GlaxoSmithKline Biologicals). Gardasil® provides protection against HPV types 6, 11, 16 and 18, while Gardasil9® protects additionally against types 31, 33, 45, 52 and 58. Cervarix® protects against HPV types 16 and 18 but does not immunise against HPV types associated with anogenital warts. For all three vaccines, two doses are recommended when vaccinating before the age of 15 years; for older ages, three doses are required.

A Cochrane review summarising the results of randomized controlled trials showed that for young women known to be HPV16/18 negative at vaccination, HPV vaccines reduced high-grade lesions (cervical intraepithelial neoplasia, CIN2 +) from 113 to 6 per 10,000 [10]. Real-world data from Australia, where vaccination rates are around 80%, due to a school-based vaccination programme at age 12 years, have shown statistically significant reductions in anogenital warts and precancerous cervical lesions in young women [11–14]. In Germany, HPV vaccination has been available since the end of 2006. From 2007 the vaccination of girls aged 12 to 17 years old was recommended and free of charge. The recommendation was changed in 2014 to girls between nine and 14 years old [15]. In 2019, 52% of 18-year-old girls in Germany were fully vaccinated [16]. Previously, two studies have evaluated the burden of HPV-associated conditions after the introduction of HPV vaccination and have reported a decrease of anogenital warts [17, 18], and severe cervical dysplasia [17]. These analyses provide a first glimpse of the potential effects of HPV vaccination, however the individual vaccination status of the study participants was not available.

We aimed to investigate HPV vaccination rates, and the effects of HPV vaccination on the risk of anogenital warts and precancerous cervical lesions in vaccinated and unvaccinated young women considering the vaccine type, and contraceptive prescription prior to vaccination in the federal state of Bavaria, Germany, a country where no organised vaccination programme has been implemented.

## Methods

### Design and setting

The retrospective cohort analyses were conducted using routine data obtained from the Bavarian Association of Statutory Health Insurance Physicians (KVB). Bavaria is the largest German state and the second most populous with about 13 million inhabitants. KVB covers 85% of the population of Bavaria, corresponding to more than 11 million people with a statutory health insurance scheme. KVB collects data quarterly from physicians on outpatient services which are eligible for remuneration. The data source allocated a unique identification code to each patient removing all personal identifiers. Our analyses were based on anonymised outpatient billing (claims) data, which contain information on sex, age, place of residence, diagnoses of anogenital warts and precancerous cervical lesions as well as drug prescription data with information on vaccination status, type of vaccine, and contraceptive prescriptions. Although HPV vaccination began in Germany in 2006, billing and prescription data were only available for analysis starting in 2008.

### Study population

**Fig. 1:**
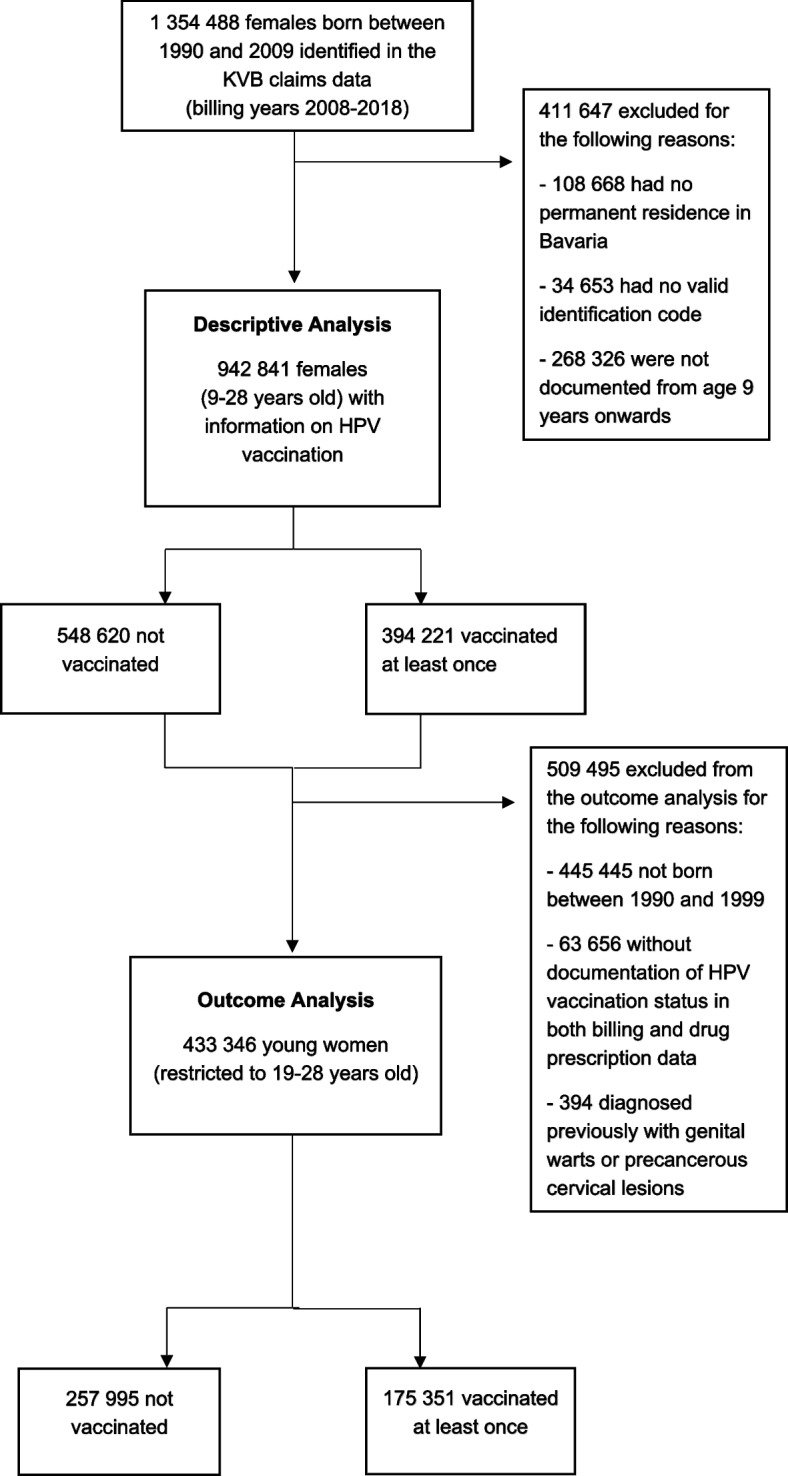
Flow chart of study population. KVB: Bavarian Association of Statutory Health Insurance Physicians; HPV: Human papillomavirus

The cohort consisted of all females born between 1990 (oldest birth cohort with documentation of HPV vaccination) and 2009 (age 9 to 28 years in 2018), permanently residing in Bavaria with a valid identification code and at least one documented visit to a physician from age nine years onwards (*n* = 942 841) (Fig. 1).

To estimate the risk of developing anogenital warts and precancerous cervical lesions (outcome analysis), we restricted the analysis population to women 19 to 28 years of age in 2018 (birth years 1990–1999), who had the HPV vaccination status documented in both billing and drug prescription data, and had not been diagnosed with anogenital warts or precancerous lesions prior to receiving the HPV vaccine (*n* = 433 346).

### HPV vaccination

All three vaccines licensed in Germany (Gardasil®, Cervarix® and Gardasil9®) were considered. Females were identified as vaccinated when an office-based physician registered in Bavaria billed an HPV vaccine according to a standard fee schedule or issued a prescription for an HPV vaccine. Age at vaccination was determined considering the respective vaccination date, and the year of birth. If the date of vaccination was not available, the quarter of the vaccination prescription was used. Females were considered fully vaccinated if they were given two doses at age nine to 14 years or three doses from age 15, respectively. Females were considered partially vaccinated if they received only one dose (age nine to 14), one or two doses (from age 15), or when the interval between the first and last dose was shorter than six months.

### Contraceptive prescription

For the use of hormonal contraceptives, women in Germany need a prescription administered by an office-based physician. The vast majority of hormonal contraceptives, especially in young women, were oral contraceptives. The variable was considered a surrogate for potential sexual initiation.

### HPV-related disease outcomes

The outcomes were diagnosis of anogenital warts (ICD-10: A63·0) or precancerous lesions of the cervix uteri, including carcinoma in situ (ICD-10: N87·1, N87·2 and D06).

### Statistical analyses

Vaccination rates by year of birth (1990–2009) were analysed descriptively. Additionally, the type of HPV vaccine administered between 2008 and 2018 was investigated.

Incidences of anogenital warts and precancerous lesions in vaccinated, partially vaccinated, and unvaccinated young women born between 1990 and 1999 were estimated, and 8-year administrative incidence for the years 2011 to 2018 were calculated. Incidence proportions were calculated by dividing the number of new cases of anogenital warts or precancerous lesions (excluding women with prior diagnoses of these diseases) with the population at risk for the respective age group.

Survival function was fitted to assess disease-free survival time to date of diagnosis of anogenital warts or precancerous lesions (age at diagnosis) for the period 2008 to 2018 in girls and young women born between 1990 and 2009. Survival probability and 95% confidence intervals (CI) were estimated, and presented as Kaplan–Meier curves, stratified for HPV vaccination status (fully, partially or not vaccinated), type of vaccine administered (Gardasil® and Gardasil9® combined versus Cervarix®), and contraceptive prescription (before first HPV vaccination).

Hazard ratios (HR) and 95% CIs were estimated for anogenital warts and precancerous lesions fitting both univariable and multivariable Cox proportional hazards models considering the time from age nine years until first diagnosis of either outcome. The proportional hazards assumption was checked visually for all variables, using the respective Kaplan–Meier curves. Analyses were performed on the analysis cohort (outcome analysis) considering HPV vaccination status (fully, partially or not vaccinated), and place of residence (25 district-free larger cities in Bavaria versus districts including smaller cities) as well as on the vaccinated females only (sensitivity analysis), considering additionally the type of vaccine administered (Gardasil® and Gardasil9® combined versus Cervarix®), and prescription of contraception prior to vaccination (yes versus no).

Statistical analyses were performed using SPSS (IBM SPSS Statistics 24) and R (Version 4.0.2).

## Results

In total, approximately 1·35 million females born between 1990 and 2009 were identified from the KVB claims data (Fig. 1). Information on HPV vaccination was available for 942 841 females and of those 394 221 were vaccinated against HPV in Bavaria between 2008 and 2018. Between 2011 and 2018, 5292 and 4385 females aged 19 to 28 years were diagnosed with anogenital warts or cervical lesions, respectively. Of those diagnosed with anogenital warts, 728, 675 and 3889 were fully, partially and not HPV vaccinated, respectively. Corresponding absolute numbers for females diagnosed with cervical lesions were 784, 1009 and 2592.

**Fig. 2:**
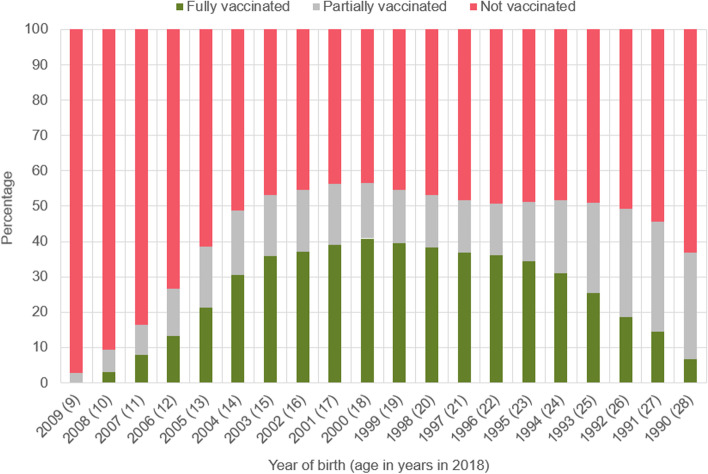
HPV vaccination rates in 2018, according to the year of birth (age in years). (*n* = 842 243; only females who visited an office-based physician in 2018)

In 2018, 34·3% of females between the ages of nine and 17 were vaccinated at least once against HPV, compared to 18·8% in 2012 and 21·1% were fully vaccinated (Additional file 1: Table S1). In 2018, 40·9% of 18-year-old women (born in 2000) and 39·1% of 17-year-olds (born in 2001) were fully vaccinated compared to only 13·3% of 12-year-old girls (born in 2006) (Fig. 2; Additional file 1: Table S9).

**Fig. 3:**
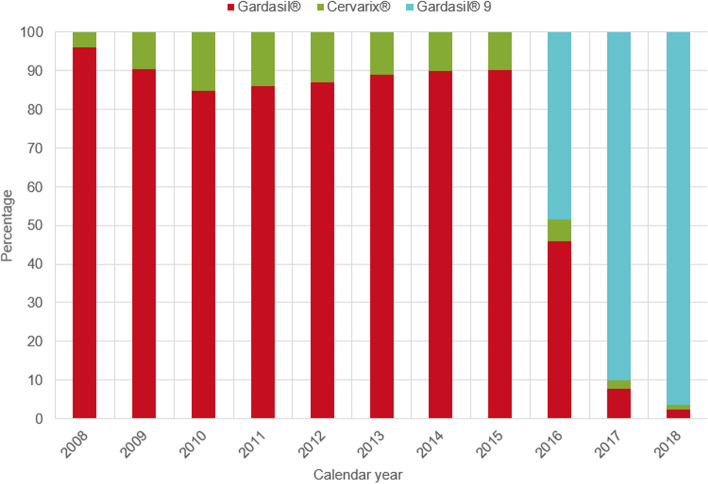
HPV vaccine type administered in Bavaria between 2008 and 2018. (*n* = 326 519; only females with available information on vaccine type)

Gardasil® was the predominantly used vaccine until 2015 (Fig. 3; Additional file 1: Table S10). In 2016, when Gardasil9® came onto the market, Gardasil® and Gardasil9® were both administered fairly evenly. From 2017 on, Gardasil 9® was the most widely used vaccine with a proportion of 96·4% in 2018. The use of Cervarix® declined from 13·9% in 2011 to 1·3% in 2018.

### Anogenital warts

The incidence of anogenital warts among unvaccinated women in the observation period of 8 years (2011–2018) was highest in women aged 24 to 28 years (2·37%, 95%CI 2·28 to 2·46) (Table 1). Partially and fully vaccinated women had a much lower incidence, with 0·84% (95%CI 0·77 to 0·91) and 0·92% (95%CI 0·83 to 1·01), respectively (Table 1).

**Table 1:** Incidence of anogenital warts and precancerous lesions in young women (2011–2018), by vaccination status

			**Incidence % (95% confidence interval)**			
**Age (years)**		**Total number of diagnoses**	**Overall**	**Not vaccinated**	**Partially vaccinated**	**Fully vaccinated**
**Anogenital warts**						
	**n**		**433 620**	**218 953**	**93 330**	**121 337**
***19***	43 565	137	0·31 (0·26 – 0·37)	0·44 (0·35 – 0·53)	0·20 (0·09 – 0·31)	0·21 (0·15 – 0·28)
***20***	44 314	224	0·51 (0·44 – 0·57)	0·72 (0·61 – 0·84)	0·35 (0·21 – 0·49)	0·30 (0·22 – 0·38)
***21***	44 809	348	0·78 (0·70 – 0·86)	1·06 (0·92 – 1·19)	0·58 (0·40– 0·77)	0·49 (0·38 – 0·60)
***22***	43 107	453	1·05 (0·96 – 1·15)	1·46 (1·30 – 1·62)	0·75 (0·54 – 0·97)	0·62 (0·50 – 0·74)
***23***	40 984	512	1·25 (1·14 – 1·36)	1·90 (1·71 – 2·09)	0·68 (0·49 – 0·88)	0·61 (0·48 – 0·74)
***24***	40 873	587	1·44 (1·32 – 1·55)	2·14 (1·93 – 2·34)	0·75 (0·56 – 0·93)	0·81 (0·65 – 0·96)
***25***	42 586	709	1·67 (1·54 – 1·79)	2·51 (2·29 – 2·72)	0·84 (0·67 – 1·02)	0·87 (0·70 – 1·05)
***26***	43 692	711	1·63 (1·51 – 1·75)	2·34 (2·14 – 2·54)	0·82 (0·67 – 0·97)	1·02 (0·80 – 1·23)
***27***	44 062	788	1·79 (1·67 – 1·91)	2·54 (2·34 – 2·73)	0·84 (0·68 – 0·99)	1·04 (0·79 – 1·29)
***28***	45 628	823	1·80 (1·68 – 1·93)	2·31 (2·14 – 2·49)	0·91 (0·75 – 1·07)	1·04 (0·68 – 1·40)
***24–28***	216 841	3618	1·67 (1·62 – 1·72)	2·37 (2·28 – 2·46)	0·84 (0·77 – 0·91)	0·92 (0·83 – 1·01)
***19–28***	433 620	5292	1·22 (1·19 – 1·25)	1·78 (1·72 – 1·83)	0·72 (0·67 – 0·78)	0·60 (0·56 – 0·64)
**Precancerous lesions**						
	**n**		**434 458**	**219 455**	**93 499**	**121 504**
***19***	43 607	78	0·18 (0·14 – 0·22)	0·17 (0·11 – 0·23)	0·28 (0·15 – 0·40)	0·15 (0·09 – 0·21)
***20***	44 355	144	0·33 (0·27 – 0·38)	0·31 (0·23 – 0·38)	0·30 (0·17 – 0·43)	0·35 (0·27 – 0·44)
***21***	44 830	188	0·42 (0·36 – 0·48)	0·44 (0·35 – 0·52)	0·40 (0·25 – 0·56)	0·41 (0·31 – 0·50)
***22***	43 143	280	0·65 (0·57 – 0·73)	0·70 (0·59 – 0·81)	0·67 (0·47 – 0·87)	0·57 (0·45 – 0·69)
***23***	41 011	347	0·85 (0·76 – 0·94)	0·87 (0·74 – 1.00)	0·80 (0·59 – 1·01)	0·84 (0·69 – 0·99)
***24***	40 913	462	1·13 (1·03 – 1·23)	1·27 (1·11 – 1·43)	1·18 (0·95 – 1·41)	0·88 (0·71 – 1·04)
***25***	42 663	547	1·28 (1·18 – 1·39)	1·46 (1·30 – 1·63)	1·27 (1·06 – 1·48)	0·94 (0·76 – 1·13)
***26***	43 811	701	1·60 (1·48 – 1·72)	1·87 (1·69 – 2·04)	1·43 (1·23 – 1·64)	1·15 (0·92 – 1·38)
***27***	44 249	761	1·72 (1·60 – 1·84)	2·01 (1·83 – 2·18)	1·46 (1·26 – 1·66)	1·19 (0·93 – 1·46)
***28***	45 876	877	1·91 (1·79 – 2·04)	2·15 (1·98 – 2·32)	1·55 (1·34 – 1·75)	1·33 (0·93 – 1·73)
***24–28***	217 512	3348	1·54 (1·49 – 1·59)	1·79 (1·72 – 1·87)	1·40 (1·31 – 1·50)	1·03 (0·93 – 1·13)
***19–28***	434 458	4385	1·01 (0·98 – 1·04)	1·18 (1·14 – 1·23)	1·08 (1·01 – 1·15)	0·65 (0·60 – 0·69)

Analysis population: Young women aged 19 to 28 years (birth year 1990 to 1999); Abbreviations: n: number of females in each vaccination category and age group; *%*: percent. Definitions: Partially vaccinated: one dose (age nine to 14), one or two doses (from age 15), or when the interval between the first and last dose was shorter than six months; Fully vaccinated: two doses at age nine to 14 years or three doses from age 15

**Fig. 4:**
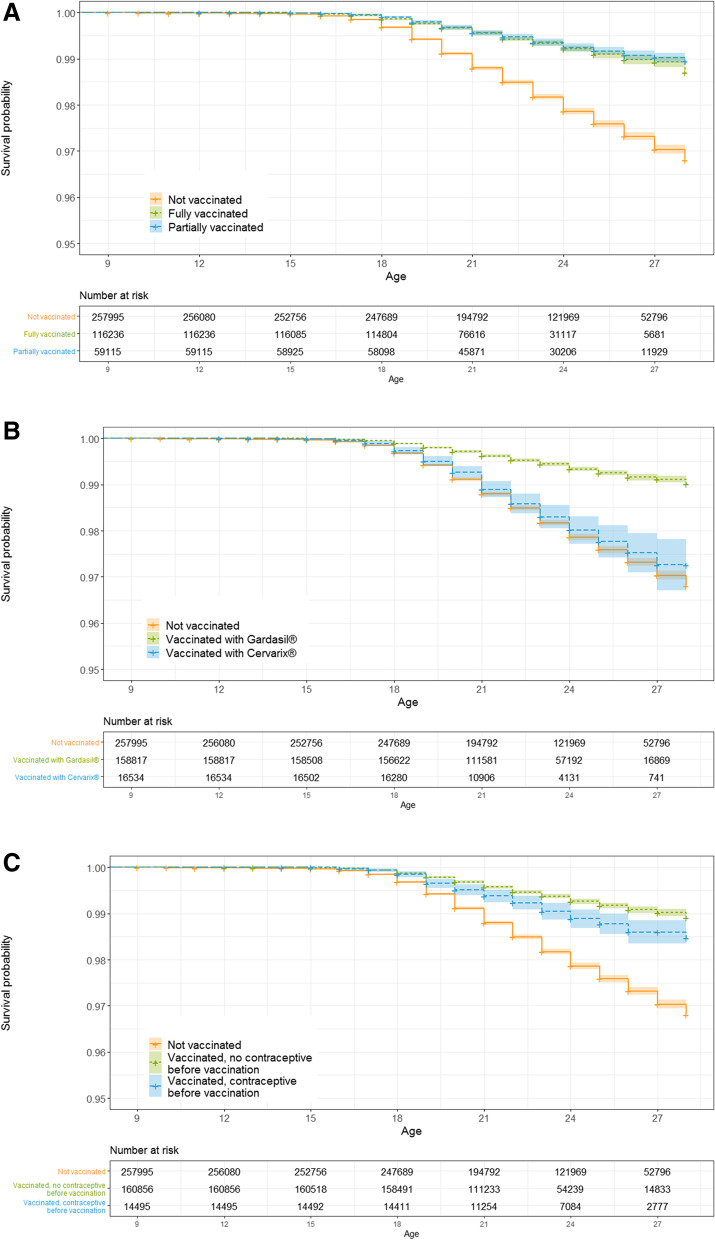
Kaplan–Meier estimates of survival function referring to time until anogenital warts diagnosis. Females aged 9 to 28 (birth years 1990–1999) years were included. The following variables were considered: **A** vaccination status, **B** vaccination status and type of vaccine administered, and **C** vaccination status and prescription of contraceptive before vaccination

The probability of being diagnosed with anogenital warts was lower in vaccinated (fully or partially) than in unvaccinated young women (Fig. 4A, Additional file 1: Table S2). The difference became apparent around the age of 18 years, however there were no differences between fully and partially vaccinated women. Young women vaccinated with Gardasil® or Gardasil9® had a lower probability of being diagnosed with anogenital warts compared to unvaccinated women or those vaccinated with Cervarix® (Fig. 4B, Additional file 1: Table S3). For vaccinated women who were prescribed contraceptives before HPV vaccination, the protective effect of HPV vaccination was smaller (Fig. 4C, Additional file 1: Table S4).

Results of multivariable Cox regression models showed that fully HPV vaccinated women had a 63% reduced risk to develop anogenital warts (adjusted HR (aHR) 0·37 (95% CI 0·34 to 0·40) (Table 2).

**Table 2:** Multivariable Cox proportional hazards models results on risk of developing anogenital warts or precancerous lesions

Covariables	Hazard ratio (95% confidence interval)	
	**Anogenital warts**	**Precancerous lesions**
**Vaccinated and unvaccinated**		
***Vaccination status***		
Not vaccinated	1 (reference)	1 (reference)
Partially vaccinated	0·34 (0·31 – 0.38)	0·81 (0·74 – 0·89)
Fully vaccinated	0·37 (0·34 – 0.40)	0·77 (0·71 – 0·84)
***Place of residence***		
Smaller city/region	1 (reference)	1 (reference)
Larger city	1·36 (1·28 – 1·45)	1·38 (1·28 – 1·48)
**Vaccinated only**		
***Vaccination status***		
Partially vaccinated	1 (reference)	1 (reference)
Fully vaccinated	1·02 (0·90 – 1·16)	0·92 (0·82 – 1·04)
***Place of residence***		
Smaller city/region	1 (reference)	1 (reference)
Larger city	1·39 (1·20 – 1·61)	1·54 (1·35 – 1·76)
***Type of vaccine***		
Vaccinated with Cervarix®	1 (reference)	1 (reference)
Vaccinated with Gardasil®/Gardasil9®	0·35 (0·30 – 0·40)	0·99 (0·80 – 1·21)
***Contraception prescription before vaccination***		
No	1 (reference)	1 (reference)
Yes	1·49 (1·25 – 1·79)	1·49 (1·27 – 1·75)

Analysis population: outcome analysis; vaccinated and unvaccinated young Bavarian women (*n* = 433 346), and vaccinated only (*n* = 175 351); HRs adjusted for all variables shown for the respective analysis. Definitions: Partially vaccinated: one dose (age nine to 14), one or two doses (from age 15), or when the interval between the first and last dose was shorter than six months; Fully vaccinated: two doses at age nine to 14 years or three doses from age 15; Place of residence: 25 district-free larger cities in Bavaria versus districts including smaller cities and rural areas

However, women living in larger cities compared to women living in smaller cities or more rural areas had an increased risk to develop anogenital warts (aHR 1·36, 95%CI 1·28 to 1·45). A similar effect was detected when restricting the analysis to vaccinated women only (aHR 1·39, 95%CI 1·20 to 1·61). In the group of vaccinated women, no difference was seen between partially and fully vaccinated women (Table 2). However, women vaccinated with Gardasil® or Gardasil9® (aHR 0·35, 95%CI 0·30 to 0·40) had a 65% lower risk to develop anogenital warts compared to women vaccinated with Cervarix®. Women with a contraceptive prescription prior to HPV vaccination had a 49% increased risk to develop anogenital warts than women with no contraceptive prescription (aHR 1·49, 95%CI 1·25 to 1·79, Table 2). The results of the crude estimates were similar for both models (Additional file 1: Table S8).

### Precancerous cervical lesions

The 8-year incidence (2011–2018) for precancerous cervical lesions in unvaccinated women in the age group 24 to 28 years was 1·79% (95%CI 1·72 to 1·87) (Table 1). The highest incidence was found in 28-year-old women with 2·15% (95%CI 1·98 to 2·32). Corresponding incidences were lower in partially and fully vaccinated women (Table 1).

**Fig. 5:**
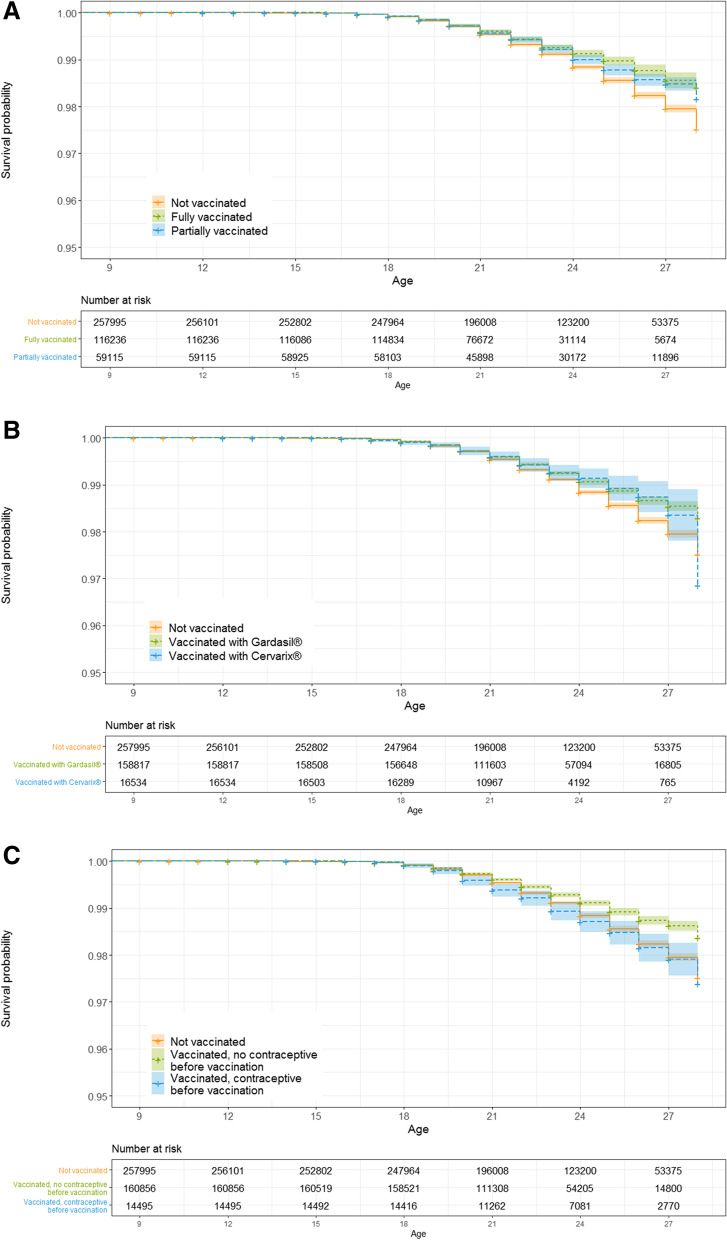
Kaplan–Meier estimates of survival function referring to time until diagnosis of precancerous cervical lesions. Analyses include carcinoma in situ. Females aged 9 to 28 (birth years 1990–1999) years were included. The following variables were considered: **A** vaccination status, **B** vaccination status and type of vaccine administered, and **C**) vaccination status and prescription of contraceptive before vaccination

The probability of being diagnosed with precancerous lesions was lower in vaccinated than in unvaccinated young women (Fig. 5A, Additional file 1: Table S5). Differences in diagnosis started to be detected after age 21 years. Incomplete vaccination status yielded slightly more diagnosis of cervical lesions. However, no differences were detected between vaccination with Gardasil®/Gardasil9® or Cervarix®. Confidence intervals for Cervarix® were larger due to the small numbers of vaccinated women (Fig. 5B, Additional file 1: Table S6). No differences were detected between unvaccinated women and those who had contraceptives prescribed before HPV vaccination, however both groups had a higher probability of being diagnosed with precancerous lesions in comparison to vaccinated women with no contraceptive prescription (Fig. 5C, Additional file 1: Table S7).

The risk to develop precancerous lesions in fully vaccinated compared to unvaccinated women was reduced by 23% (aHR 0·77, 95%CI 0·71 to 0·84) (Table 2). The sensitivity analysis of vaccinated women only revealed a non-significant difference between partially vaccinated and fully vaccinated women (aHR 0·92, 95%CI 0·82 to 1·04) (Table 2). Vaccinated women who live in larger cities had a 54% increased risk compared to women living in smaller cities and more rural regions (aHR 1·54, 95%CI 1·35 to 1·76). No differences were detected regarding the type of vaccine administered. Vaccinated women with a contraceptive prescription prior to HPV vaccination had a 49% increased risk to develop precancerous cervical lesions (aHR 1·49, 95%CI 1·27 to 1·75, Table 2) compared to those without it. The results of the unadjusted HR were similar for both models (Additional file 1: Table S8).

## Discussion

Only 13·3% of 12-year-olds and 40·9% of 18-year-olds were fully vaccinated in 2018 in Bavaria, with 96·4% of all vaccinated girls receiving Gardasil® or Gardasil9®. The incidences of anogenital warts and precancerous lesions in vaccinated women were lower than in unvaccinated. Vaccinated women had a 63% and 23% lower risk than unvaccinated women for the development of anogenital warts and precancerous cervical lesions, respectively. However, residing in a larger city and being prescribed contraceptives prior to vaccination counteracted the protective effects.

The vaccination rates in Germany are among the lowest in Europe [19]. Germany has not implemented an organised vaccination programme. In Australia, five years after the implementation of an organised school-based HPV vaccination programme in 2007, 73% of 12 to 13-year-olds were fully vaccinated [11]. In Bavaria, 11 years after the recommendation of the HPV vaccination in 2007, only 13·3% of 12-year-old girls were fully vaccinated. Girls receive the vaccine free of charge if they visit an office-based paediatrician, gynaecologist or general practitioner, who recommends the HPV vaccination and chooses the vaccine. The role of the office-based physicians in HPV vaccination in Germany seems crucial, since two-thirds of unvaccinated girls had frequent visits in these practices [20]. Other reasons for not getting vaccinated might be parental concerns and negative beliefs towards vaccines in general [21]. The low vaccination rates in younger age groups might indicate parents’ preference to vaccinate children later than the recommended age of 9 to 14 years old.

No differences in the development of anogenital warts were detected between fully vaccinated and partially vaccinated women. For cervical lesions, only small differences were noted. Evidence from a recent systematic review showed that the protection against HPV infections was not significantly associated with the number of doses for up to seven years of follow-up [22]. Vaccination with Gardasil®/Gardasil9® compared to Cervarix® showed similar results in the reduction of precancerous cervical lesions. This is consistent with findings in other countries [23]. As expected, we did not find any differences between women vaccinated with Cervarix® and unvaccinated women regarding the diagnosis of anogenital warts.

Prior studies in countries with high vaccination rates have reported reduced risks for anogenital warts and precancerous lesions among HPV vaccinated women [11–14]. Within five years after HPV vaccine introduction, the risk of developing high grade lesions was reduced by 39% in fully vaccinated young women in Australia [12]. Regarding anogenital warts, reductions of up to 45% were reported after seven years [14, 23]. We found similar reductions in these HPV-related diseases in our study after 11 years of follow-up.

The higher risk among vaccinated women living in larger cities in comparison to smaller cities and rural regions might be due to more frequent visits to office-based gyneacologists, related to an easier access to healthcare services, resulting in more diagnoses. Other factors might be earlier sexual initiation or more sexual partners in women living in larger cities in comparison to rural areas, however no data was available on behavioural factors in our study.

In Germany, females need a prescription by an office-based physician to purchase hormonal contraceptives. Most visit a gynaecologist, where they routinely receive a medical exam and a cytological screening with the Pap smear. A contraception prescription could be an indicator of girls being sexually active before HPV vaccination, and therefore no longer HPV naïve. This might explain the increased risk of developing anogenital warts and precancerous cervical lesions among vaccinated girls who had been prescribed contraceptives prior to HPV vaccination and further underlines that the HPV vaccine effectiveness is highest when administered before first sexual activity [10].

### Strengths and limitations

This is one of the first studies to provide evidence of the effectiveness of HPV vaccination in the prevention of anogenital warts and precancerous lesions in a country with low vaccination rates. We used comprehensive, routinely collected claims data on females with statutory health insurance. We used both outpatient claims and drug prescription data which allowed population-based outcome estimations based on vaccination status, type of vaccine administered and contraception prescription. Our sample is a representative cohort of girls and young women and the results are generalisable.

However, the usage of claims data has some limitations. The diagnoses are based on ICD-10 coding from office-based physicians, which could vary in quality and accuracy [24]. Females who did not visit an office-based physician were not documented even if anogenital warts or cervical lesions occurred, which could have underestimated the incidence of the investigated outcomes. Information on potential confounders, such as education or socio-economic status were not available. We restricted the outcome analysis to women who had additional information on HPV vaccination status in the drug prescription data. This might slightly overestimate the effect of HPV vaccination on both outcomes. However, it increases the robustness of our results.

Lastly, we assumed that the prescription of hormonal contraceptives was an indicator of sexual activity. Nevertheless, not all females who are sexually active utilize hormonal contraception, nor all females who take hormonal contraception have initiated sexual activity. Additionally, hormonal contraception does not fully predict sexual behaviour and it can be prescribed also for other indications, such as irregular menstrual cycles.

## Conclusions

There were marked differences between vaccinated, and unvaccinated girls and women in terms of incidence of anogenital warts, and precancerous lesions of the cervix uteri, despite low vaccination rates. An increase in vaccination rates could be achieved through the implementation of an organised vaccination programme. Gender-neutral approaches and high vaccination rates are expected to contribute to the elimination of cervical cancer as targeted by WHO and to the reduction of other HPV-attributable diseases.

## Supplementary Information

**Additional file 1: Table S1.** HPV vaccination rates among 12-, 18- and 9 to 17- year-old females during 2012-2018. **Table S2.** Kaplan-Meier estimates of survival function referring to time until anogenital warts diagnosis by vaccination status. **Table S3.** Kaplan-Meier estimates of survival function referring to time until anogenital warts diagnosis by vaccination status and vaccine type. **Table S4.** Kaplan-Meier estimates of survival function referring to time until anogenital warts diagnosis by vaccination status and contraceptive prescription. **Table S5.** Kaplan-Meier estimates of survival function referring to time until cervical lesions diagnosis by vaccination status. **Table S6.** Kaplan-Meier estimates of survival function referring to time until cervical lesions diagnosis by vaccination status and vaccine type. **Table S7.** Kaplan-Meier estimates of survival function referring to time until cervical lesions diagnosis by vaccination status and contraceptive prescription. **Table S8.** Univariable Cox regression model results regarding risk to develop genital warts or precancerous lesions in young women. **Table S9.** HPV vaccination rates in 2018, according to the year of birth (age in years). **Table S10.** HPV vaccine type administered in Bavaria between 2008 and 2018.

## Data Availability

The data that support the findings of this study are available from the KVB but restrictions apply to the availability of these data. Data are however available from the authors upon reasonable request and with permission of the KVB.

## Abbreviations

HPV: Human papillomavirus
KVB: The Bavarian Association of Statutory Health Insurance Physicians
HR: Hazard Ratio
hrHPV: High-risk human papillomavirus
lrHPV: Low-risk human papillomavirus
CI: Confidence Interval
FDA: Food and Drug Administration
EMA: European Medicines Agency
CIN2 +: Cervical Intraepithelial Neoplasia 2 +
aHR: Adjusted Hazard Ratio
WHO: World Health Organization
