# Supplementary material for: HPV vaccination leads to decrease of anogenital warts and precancerous lesions of the cervix uteri in young women with low vaccination rates: a retrospective cohort analysis
Source: BMC Cancer. 2022 Dec 9;22:1293. doi: 10.1186/s12885-022-10214-1 (PMC9732997; doi:10.1186/s12885-022-10214-1)
Supplement: Supplementary file 1 — Additional file 1: Table S1. HPV vaccination rates among 12-, 18- and 9 to 17- year-old females during 2012-2018. Table S2. Kaplan-Meier estimates of survival function referring to time until anogenital warts diagnosis by vaccination status. Table S3. Kaplan-Meier estimates of survival function referring to time until anogenital warts diagnosis by vaccination status and vaccine type. Table S4. Kaplan-Meier estimates of survival function referring to time until anogenital warts diagnosis by vaccination status and contraceptive prescription. Table S5. Kaplan-Meier estimates of survival function referring to time until cervical lesions diagnosis by vaccination status. Table S6. Kaplan-Meier estimates of survival function referring to time until cervical lesions diagnosis by vaccination status and vaccine type. Table S7. Kaplan-Meier estimates of survival function referring to time until cervical lesions diagnosis by vaccination status and contraceptive prescription. Table S8. Univariable Cox regression model results regarding risk to develop genital warts or precancerous lesions in young women. Table S9. HPV vaccination rates in 2018, according to the year of birth (age in years). Table S10. HPV vaccine type administered in Bavaria between 2008 and 2018. [file 12885_2022_10214_MOESM1_ESM.pdf]

Osmani et al. Supplementary tables:
HPV vaccination leads to decrease of anogenital warts and precancerous lesions of the cervix uteri in young women with low vaccination rates: a retrospective cohort analysis

Additional file 1

Table S1. HPV vaccination rates among 12-, 18- and 9 to 17- year-old females during 2012-2018

**Year**	**2012**		**2014**		**2016**		**2018**	
	**n**	**%**	**n**	**%**	**n**	**%**	**n**	**%**
12-year-old females								
Not vaccinated	41 466	95·8	38 343	94·4	32 858	82·3	27 845	73·4
Partially vaccinated	1 584	3·7	2 036	5·0	4 299	10·8	5 071	13·4
Fully vaccinated	223	0·5	245	0·6	2 750	6·9	5 038	13·3
Total	43 273	100	40 624	100	39 907	100	37 954	100
18 year-old females								
Not vaccinated	21 447	48·6	22 247	49·2	21 213	46·6	19 776	43·4
Partially vaccinated	9 310	21·1	6 991	15·5	7 126	15·7	7 154	15·7
Fully vaccinated	13 389	30·3	16 010	35·4	17 183	37·7	18 602	40·9
Total	44 146	100	45 248	100	45 522	100	45 532	100
9 to 17-year-old females								
Not vaccinated	313 536	81·2	301 511	79·3	267 220	72·2	237 832	65·7
Partially vaccinated	31 819	8·2	32 966	8·7	41 319	11·2	47 614	13·2
Fully vaccinated	40 936	10·6	45 544	12·0	61 344	16·6	76 548	21·2
Total	386 291	100	380 021	100	369 883	100	361 994	100

Abbreviations: n: total number of females in the age group by year; %: percent. Definitions: Partially vaccinated: one dose (age nine to 14), one or two doses (from age 15), or when the interval between the first and last dose was shorter than six months; Fully vaccinated: two doses at age nine to 14 years or three doses from age 15. Due to approximation, the totals do not necessarily add up to 100%.

Osmani et al. Supplementary tables:

HPV vaccination leads to decrease of anogenital warts and precancerous lesions of the cervix uteri in young women with low vaccination rates: a retrospective cohort analysis

Table S2. Kaplan-Meier estimates of survival function referring to time until anogenital warts diagnosis by vaccination status

Age (years)	Survival probability (95% confidence interval)		
	**Not vaccinated**	**Partially vaccinated**	**Fully vaccinated**
**9**	1·000 (1·000 – 1·000)	1·000 (1·000 – 1.000)	1·000 (1·000 – 1·000)
**10**	1·000 (1·000 – 1·000)	1·000 (1·000 – 1·000)	1·000 (1·000 – 1·000)
**11**	1·000 (1·000 – 1·000)	1·000 (1·000 – 1·000)	1·000 (1·000 – 1·000)
**12**	1·000 (1·000 – 1·000)	1·000 (1·000 – 1·000)	1·000 (1·000 – 1·000)
**13**	1·000 (1·000 – 1·000)	1·000 (1·000 – 1·000)	1·000 (1·000 – 1·000)
**14**	1·000 (1·000 – 1·000)	1·000 (1·000 – 1·000)	1·000 (1·000 – 1·000)
**15**	1·000 (1·000 – 1·000)	1·000 (1·000 – 1·000)	1·000 (1·000 – 1·000)
**16**	0·999 (0·999 – 0·999)	1·000 (1·000 – 1·000)	1·000 (1·000 – 1·000)
**17**	0·999 (0·998 – 0·999)	1·000 (0·999 – 1·000)	0·999 (0·999 – 0·999)
**18**	0·997 (0·997 – 0·997)	0·999 (0·999 – 0·999)	0·999 (0·998 – 0·999)
**19**	0·994 (0·994 – 0·995)	0·998 (0·998 – 0·998)	0·998 (0·997 – 0·998)
**20**	0·991 (0·991 – 0·992)	0·997 (0·996 – 0·997)	0·997 (0·996 – 0·997)
**21**	0·988 (0·988 – 0·988)	0·996 (0·995 – 0·996)	0·996 (0·995 – 0·996)
**22**	0·985 (0·984 – 0·985)	0·995 (0·994 – 0·995)	0·994 (0·994 – 0·995)
**23**	0·982 (0·981 – 0·982)	0·994 (0·993 – 0·994)	0·993 (0·993 – 0·994)
**24**	0·979 (0·978 – 0·979)	0·992 (0·992 – 0·993)	0·992 (0·991 – 0·993)
**25**	0·976 (0·975 – 0·977)	0·992 (0·991 – 0·993)	0·991 (0·990 – 0·992)
**26**	0·973 (0·972 – 0·974)	0·991 (0·990 – 0·992)	0·990 (0·989 – 0·991)
**27**	0·970 (0·969 – 0·971)	0·990 (0·989 – 0·991)	0·989 (0·988 – 0·990)
**28**	0·968 (0·967 – 0·969)	0·990 (0·988 – 0·991)	0·987 (0·984 – 0·990)

Included: Young Bavarian women aged 9 to 28 (birth years 1990-2009).

Osmani et al. Supplementary tables:
HPV vaccination leads to decrease of anogenital warts and precancerous lesions of the cervix uteri in young women with low vaccination rates: a retrospective cohort analysis

Table S3. Kaplan-Meier estimates of survival function referring to time until anogenital warts diagnosis by vaccination status and vaccine type

Age (years)	Survival probability (95% confidence interval)		
	**Not vaccinated**	**Vaccinated with Gardasil®**	**Vaccinated with Cervarix®**
**9**	1·000 (1·000 – 1·000)	1·000 (1·000 – 1·000)	1·000 (1·000 – 1·000)
**10**	1·000 (1·000 – 1·000)	1·000 (1·000 – 1·000)	1·000 (1·000 – 1·000)
**11**	1·000 (1·000 – 1·000)	1·000 (1·000 – 1·000)	1·000 (1·000 – 1·000)
**12**	1·000 (1·000 – 1·000)	1·000 (1·000 – 1·000)	1·000 (1·000 – 1·000)
**13**	1·000 (1·000 – 1·000)	1·000 (1·000 – 1·000)	1·000 (1·000 – 1·000)
**14**	1·000 (1·000 – 1·000)	1·000 (1·000 – 1·000)	1·000 (1·000 – 1·000)
**15**	1·000 (1·000 – 1·000)	1·000 (1·000 – 1·000)	1·000 (1·000 – 1·000)
**16**	0·999 (0·999 – 0·999)	1·000 (1·000 – 1·000)	1·000 (0·999 – 1·000)
**17**	0·999 (0·998 – 0·999)	0·999 (0·999 – 1·000)	0·999 (0·998 – 0·999)
**18**	0·997 (0·997 – 0·997)	0·999 (0·999 – 0·999)	0·997 (0·997 – 0·998)
**19**	0·994 (0·994 – 0·995)	0·998 (0·998 – 0·998)	0·995 (0·994 – 0·996)
**20**	0·991 (0·991 – 0·992)	0·997 (0·997 – 0·997)	0·993 (0·991 – 0·994)
**21**	0·988 (0·988 – 0·988)	0·996 (0·996 – 0·997)	0·989 (0·987 – 0·991)
**22**	0·985 (0·984 – 0·985)	0·995 (0·995 – 0·996)	0·986 (0·984 – 0·988)
**23**	0·982 (0·981 – 0·982)	0·994 (0·994 – 0·995)	0·983 (0·981 – 0·986)
**24**	0·979 (0·978 – 0·979)	0·993 (0·993 – 0·994)	0·980 (0·977 – 0·983)
**25**	0·976 (0·975 – 0·977)	0·993 (0·992 – 0·993)	0·978 (0·974 – 0·981)
**26**	0·973 (0·972 – 0·974)	0·992 (0·991 – 0·992)	0·975 (0·971 – 0·979)
**27**	0·970 (0·969 – 0·971)	0·991 (0·990 – 0·992)	0·973 (0·967 – 0·978)
**28**	0·968 (0·967 – 0·969)	0·990 (0·989 – 0·991)	0·973 (0·967 – 0·978)

Included: Young Bavarian women aged 9 to 28 (birth years 1990-2009).

Osmani et al. Supplementary tables:
HPV vaccination leads to decrease of anogenital warts and precancerous lesions of the cervix uteri in young women with low vaccination rates: a retrospective cohort analysis

Table S4. Kaplan-Meier estimates of survival function referring to time until anogenital warts diagnosis by vaccination status and contraceptive prescription

Age (years)	Survival probability (95% confidence interval)		
	**Not vaccinated**	**Vaccinated, no contraceptive before vaccination**	**Vaccinated, contraceptive before vaccination**
**9**	1·000 (1·000 – 1·000)	1·000 (1·000 – 1·000)	1·000 (1·000 – 1·000)
**10**	1·000 (1·000 – 1·000)	1·000 (1·000 – 1·000)	1·000 (1·000 – 1·000)
**11**	1·000 (1·000 – 1·000)	1·000 (1·000 – 1·000)	1·000 (1·000 – 1·000)
**12**	1·000 (1·000 – 1·000)	1·000 (1·000 – 1·000)	1·000 (1·000 – 1·000)
**13**	1·000 (1·000 – 1·000)	1·000 (1·000 – 1·000)	1·000 (1·000 – 1·000)
**14**	1·000 (1·000 – 1·000)	1·000 (1·000 – 1·000)	1·000 (1·000 – 1·000)
**15**	1·000 (1·000 – 1·000)	1·000 (1·000 – 1·000)	1·000 (1·000 – 1·000)
**16**	0·999 (0·999 – 0·999)	1·000 (1·000 – 1·000)	1·000 (0·999 – 1·000)
**17**	0·999 (0·998 – 0·999)	0·999 (0·999 – 1·000)	0·999 (0·999 – 1·000)
**18**	0·997 (0·997 – 0·997)	0·999 (0·999 – 0·999)	0·998 (0·998 – 0·999)
**19**	0·994 (0·994 – 0·995)	0·998 (0·998 – 0·998)	0·997 (0·996 – 0·997)
**20**	0·991 (0·991 – 0·992)	0·997 (0·997 – 0·997)	0·995 (0·994 – 0·996)
**21**	0·988 (0·988 – 0·988)	0·996 (0·995 – 0·996)	0·994 (0·992 – 0·995)
**22**	0·985 (0·984 – 0·985)	0·995 (0·994 – 0·995)	0·992 (0·991 – 0·994)
**23**	0·982 (0·981 – 0·982)	0·994 (0·993 – 0·994)	0·990 (0·989 – 0·992)
**24**	0·979 (0·978 – 0·979)	0·993 (0·992 – 0·993)	0·989 (0·987 – 0·991)
**25**	0·976 (0·975 – 0·977)	0·992 (0·991 – 0·992)	0·988 (0·986 – 0·990)
**26**	0·973 (0·972 – 0·974)	0·991 (0·990 – 0·992)	0·986 (0·984 – 0·989)
**27**	0·970 (0·969 – 0·971)	0·990 (0·989 – 0·991)	0·986 (0·984 – 0·989)
**28**	0·968 (0·967 – 0·969)	0·989 (0·988 – 0·990)	0·985 (0·981 – 0·988)

Included: Young Bavarian women aged 9 to 28 (birth years 1990-2009).

Osmani et al. Supplementary tables:
HPV vaccination leads to decrease of anogenital warts and precancerous lesions of the cervix uteri in young women with low vaccination rates: a retrospective cohort analysis

Table S5. Kaplan-Meier estimates of survival function referring to time until cervical lesions diagnosis by vaccination status

Age (years)	Survival probability (95% confidence interval)		
	**Not vaccinated**	**Partially vaccinated**	**Fully vaccinated**
**9**	1·000 (1·000 – 1·000)	1·000 (1·000 – 1·000)	1·000 (1·000 – 1·000)
**10**	1·000 (1·000 – 1·000)	1·000 (1·000 – 1·000)	1·000 (1·000 – 1·000)
**11**	1·000 (1·000 – 1·000)	1·000 (1·000 – 1·000)	1·000 (1·000 – 1·000)
**12**	1·000 (1·000 – 1·000)	1·000 (1·000 – 1·000)	1·000 (1·000 – 1·000)
**13**	1·000 (1·000 – 1·000)	1·000 (1·000 – 1·000)	1·000 (1·000 – 1·000)
**14**	1·000 (1·000 – 1·000)	1·000 (1·000 – 1·000)	1·000 (1·000 – 1·000)
**15**	1·000 (1·000 – 1·000)	1·000 (1·000 – 1·000)	1·000 (1·000 – 1·000)
**16**	1·000 (1·000 – 1·000)	1·000 (1·000 – 1·000)	1·000 (1·000 – 1·000)
**17**	1·000 (1·000 – 1·000)	1·000 (1·000 – 1·000)	1·000 (1·000 – 1·000)
**18**	0·999 (0·999 – 0·999)	0·999 (0·999 – 0·999)	0·999 (0·999 – 0·999)
**19**	0·998 (0·998 – 0·998)	0·998 (0·998 – 0·999)	0·998 (0·998 – 0·999)
**20**	0·997 (0·997 – 0·997)	0·997 (0·997 – 0·998)	0·997 (0·997 – 0·998)
**21**	0·995 (0·995 – 0·996)	0·996 (0·995 – 0·996)	0·996 (0·996 – 0·996)
**22**	0·993 (0·993 – 0·994)	0·994 (0·994 – 0·995)	0·994 (0·994 – 0·995)
**23**	0·991 (0·991 – 0·992)	0·992 (0·991 – 0·993)	0·993 (0·992 – 0·993)
**24**	0·988 (0·988 – 0·989)	0·990 (0·989 – 0·991)	0·991 (0·991 – 0·992)
**25**	0·986 (0·985 – 0·986)	0·988 (0·987 – 0·989)	0·990 (0·989 – 0·991)
**26**	0·982 (0·982 – 0·983)	0·986 (0·984 – 0·987)	0·988 (0·986 – 0·989)
**27**	0·980 (0·979 – 0·980)	0·985 (0·983 – 0·986)	0·986 (0·984 – 0·987)
**28**	0·975 (0·974 – 0·976)	0·982 (0·980 – 0·984)	0·984 (0·981 – 0·987)

Included: Young Bavarian women aged 9 to 28 (birth years 1990-2009).

Osmani et al. Supplementary tables:

HPV vaccination leads to decrease of anogenital warts and precancerous lesions of the cervix uteri in young women with low vaccination rates: a retrospective cohort analysis

Table S6. Kaplan-Meier estimates of survival function referring to time until cervical lesions diagnosis by vaccination status and vaccine type

Age (years)	Survival probability (95% confidence interval)		
	**Not vaccinated**	**Vaccinated with Gardasil®**	**Vaccinated with Cervarix®**
**9**	1·000 (1·000 – 1·000)	1·000 (1·000 – 1·000)	1·000 (1·000 – 1·000)
**10**	1·000 (1·000 – 1·000)	1·000 (1·000 – 1·000)	1·000 (1·000 – 1·000)
**11**	1·000 (1·000 – 1·000)	1·000 (1·000 – 1·000)	1·000 (1·000 – 1·000)
**12**	1·000 (1·000 – 1·000)	1·000 (1·000 – 1·000)	1·000 (1·000 – 1·000)
**13**	1·000 (1·000 – 1·000)	1·000 (1·000 – 1·000)	1·000 (1·000 – 1·000)
**14**	1·000 (1·000 – 1·000)	1·000 (1·000 – 1·000)	1·000 (1·000 – 1·000)
**15**	1·000 (1·000 – 1·000)	1·000 (1·000 – 1·000)	1·000 (1·000 – 1·000)
**16**	1·000 (1·000 – 1·000)	1·000 (1·000 – 1·000)	1·000 (1·000 – 1·000)
**17**	1·000 (1·000 – 1·000)	1·000 (1·000 – 1·000)	0·999 (0·999 – 1·000)
**18**	0·999 (0·999 – 0·999)	0·999 (0·999 – 0·999)	0·999 (0·998 – 0·999)
**19**	0·998 (0·998 – 0·998)	0·998 (0·998 – 0·999)	0·998 (0·998 – 0·999)
**20**	0·997 (0·997 – 0·997)	0·997 (0·997 – 0·997)	0·997 (0·996 – 0·998)
**21**	0·995 (0·995 – 0·996)	0·996 (0·996 – 0·996)	0·996 (0·995 – 0·997)
**22**	0·993 (0·993 – 0·994)	0·994 (0·994 – 0·995)	0·994 (0·993 – 0·996)
**23**	0·991 (0·991 – 0·992)	0·992 (0·992 – 0·993)	0·993 (0·991 – 0·994)
**24**	0·988 (0·988 – 0·989)	0·991 (0·990 – 0·991)	0·991 (0·989 – 0·993)
**25**	0·986 (0·985 – 0·986)	0·989 (0·988 – 0·989)	0·989 (0·987 – 0·992)
**26**	0·982 (0·982 – 0·983)	0·987 (0·986 – 0·988)	0·987 (0·984 – 0·991)
**27**	0·980 (0·979 – 0·980)	0·985 (0·984 – 0·986)	0·984 (0·978 – 0·989)
**28**	0·975 (0·974 – 0·976)	0·983 (0·981 – 0·985)	0·969 (0·951 – 0·986)

Included: Young Bavarian women aged 9 to 28 (birth years 1990-2009).

Osmani et al. Supplementary tables:

HPV vaccination leads to decrease of anogenital warts and precancerous lesions of the cervix uteri in young women with low vaccination rates: a retrospective cohort analysis

Table S7. Kaplan-Meier estimates of survival function referring to time until cervical lesions diagnosis by vaccination status and contraceptive prescription

Age (years)	Survival probability (95% confidence interval)		
	Not vaccinated	Vaccinated, no contraceptive before vaccination	Vaccinated, contraceptive before vaccination
**9**	1·000 (1·000 – 1·000)	1·000 (1·000 – 1·000)	1·000 (1·000 – 1·000)
**10**	1·000 (1·000 – 1·000)	1·000 (1·000 – 1·000)	1·000 (1·000 – 1·000)
**11**	1·000 (1·000 – 1·000)	1·000 (1·000 – 1·000)	1·000 (1·000 – 1·000)
**12**	1·000 (1·000 – 1·000)	1·000 (1·000 – 1·000)	1·000 (1·000 – 1·000)
**13**	1·000 (1·000 – 1·000)	1·000 (1·000 – 1·000)	1·000 (1·000 – 1·000)
**14**	1·000 (1·000 – 1·000)	1·000 (1·000 – 1·000)	1·000 (1·000 – 1·000)
**15**	1·000 (1·000 – 1·000)	1·000 (1·000 – 1·000)	1·000 (1·000 – 1·000)
**16**	1·000 (1·000 – 1·000)	1·000 (1·000 – 1·000)	1·000 (1·000 – 1·000)
**17**	1·000 (1·000 – 1·000)	1·000 (1·000 – 1·000)	1·000 (1·000 – 1·000)
**18**	0·999 (0·999 – 0·999)	0·999 (0·999 – 0·999)	0·999 (0·999 – 1·000)
**19**	0·998 (0·998 – 0·998)	0·999 (0·998 – 0·999)	0·998 (0·997 – 0·999)
**20**	0·997 (0·997 – 0·997)	0·997 (0·997 – 0·998)	0·996 (0·995 – 0·997)
**21**	0·995 (0·995 – 0·996)	0·996 (0·996 – 0·996)	0·994 (0·992 – 0·995)
**22**	0·993 (0·993 – 0·994)	0·995 (0·994 – 0·995)	0·992 (0·991 – 0·994)
**23**	0·991 (0·991 – 0·992)	0·993 (0·992 – 0·993)	0·989 (0·987 – 0·991)
**24**	0·988 (0·988 – 0·989)	0·991 (0·990 – 0·992)	0·987 (0·985 – 0·989)
**25**	0·986 (0·985 – 0·986)	0·989 (0·988 – 0·990)	0·985 (0·982 – 0·987)
**26**	0·982 (0·982 – 0·983)	0·987 (0·986 – 0·988)	0·982 (0·979 – 0·985)
**27**	0·980 (0·979 – 0·980)	0·986 (0·985 – 0·987)	0·979 (0·976 – 0·983)
**28**	0·975 (0·974 – 0·976)	0·984 (0·982 – 0·985)	0·974 (0·968 – 0·980)

Included: Young Bavarian women aged 9 to 28 (birth years 1990-2009).

Table S8. Univariable Cox regression model results regarding risk to develop genital warts or precancerous lesions in young women

Covariables	Hazard ratio (95% confidence interval)	
	Genital warts	Precancerous lesions
**Vaccinated and unvaccinated**		
Vaccination status		
Not vaccinated	1 (reference)	1 (reference)
Partially vaccinated	0·34 (0·31 – 0·38)	0·81 (0·74 – 0·89)
Fully vaccinated	0·37 (0·34 – 0.40)	0·77 (0·70 – 0·83)
**Vaccinated only**		
Vaccination status		
Partially vaccinated	1 (reference)	1 (reference)
Fully vaccinated	1·08 (0·95 – 1·22)	0·91 (0·81 – 1·02)

Note: Analysis population: outcome analysis; vaccinated and unvaccinated (n=433 346), and vaccinated only (n=175 351). Definitions: Partially vaccinated: one dose (age nine to 14), one or two doses (from age 15), or when the interval between the first and last dose was shorter than six months; Fully vaccinated: two doses at age nine to 14 years or three doses from age 15.

Osmani et al. Supplementary tables:
HPV vaccination leads to decrease of anogenital warts and precancerous lesions of the cervix uteri in young women with low vaccination rates: a retrospective cohort analysis

Table S9. HPV vaccination rates in 2018, according to the year of birth (age in years)

Year of birth (Age in 2018)	Fully vaccinated		Partially vaccinated		Not vaccinated	
	N	%	N	%	N	%
**1990 (28)**	3 095	6·7	13 861	30·2	29 016	63·1
**1991 (27)**	6 375	14·4	13 822	31·2	24 093	54·4
**1992 (26)**	8 200	18·7	13 343	30·4	22 300	50·9
**1993 (25)**	10 828	25·4	10 944	25·6	20 915	49·0
**1994 (24)**	12 669	31·0	8 473	20·7	19 782	48·3
**1995 (23)**	14 128	34·4	6 897	16·8	19 997	48·7
**1996 (22)**	15 540	36·0	6 393	14·8	21 221	49·2
**1997 (21)**	16 542	36·9	6 687	14·9	21 614	48·2
**1998 (20)**	16 946	38·2	6 656	15·0	20 766	46·8
**1999 (19)**	17 247	39·5	6 519	14·9	19 848	45·5
**2000 (18)**	18 602	40·9	7 154	15·7	19 776	43·4
**2001 (17)**	16 844	39·1	7 422	17·2	18 836	43·7
**2002 (16)**	15 566	37·1	7 317	17·5	19 018	45·4
**2003 (15)**	14 527	35·9	6 944	17·2	18 998	46·9
**2004 (14)**	12 030	30·4	7 286	18·4	20 219	51·1
**2005 (13)**	8 068	21·2	6 663	17·5	23 375	61·3
**2006 (12)**	5 038	13·3	5 071	13·4	27 845	73·4
**2007 (11)**	3 142	7·9	3 384	8·5	33 171	83·6
**2008 (10)**	1 241	3·1	2 515	6·2	36 915	90·8
**2009 (9)**	92	0·2	1 012	2·5	39 455	97·3
**Overall**	216 720	25·7	148 363	17·6	477 160	56·7

Note: n= 842 243; only females who visited an office-based physician in 2018

Osmani et al. Supplementary tables:
HPV vaccination leads to decrease of anogenital warts and precancerous lesions of the cervix uteri in young women with low vaccination rates: a retrospective cohort analysis

Table S10. HPV vaccine type administered in Bavaria between 2008 and 2018

**Calendar year**	**Total**	**Vaccine type**					
		**Gardasil®**		**Cervarix®**		**Gardasil9®**	
		N	%	N	%	N	%
**2008**	29 696	28 511	96·0	1 185	4·0	0	0·0
**2009**	37 374	33 778	90·4	3 596	9·6	0	0·0
**2010**	15 198	12 894	84·8	2 304	15·2	0	0·0
**2011**	19 728	16 976	86·1	2 752	13·9	0	0·0
**2012**	24 814	21 595	87·0	3 219	13·0	0	0·0
**2013**	24 843	22 107	89·0	2 736	11·0	0	0·0
**2014**	28 138	25 299	89·9	2 839	10·1	0	0·0
**2015**	36 617	33 055	90·3	3 562	9·7	0	0·0
**2016**	37 803	17 351	45·9	2 157	5·7	18 295	48·4
**2017**	35 609	2 710	7·6	784	2·2	32 115	90·2
**2018**	36 699	862	2·3	476	1·3	35 361	96·4
**Overall**	326 519	215 138	65·9	25 610	7·8	85 771	26·3

Note: n= 326 519; only females with available information on vaccine type; Total: Number of individuals with information on HPV vaccine type.
